# Peripheral Blood Cells from Patients with Autoimmune Addison's Disease Poorly Respond to Interferons *In Vitro*, Despite Elevated Serum Levels of Interferon-Inducible Chemokines

**DOI:** 10.1089/jir.2014.0171

**Published:** 2015-10-01

**Authors:** Kine Edvardsen, Trine Bjånesøy, Alexander Hellesen, Lars Breivik, Marit Bakke, Eystein S. Husebye, Eirik Bratland

**Affiliations:** ^1^ Department of Clinical Science, University of Bergen, Bergen, Norway.; ^2^ Department of Biomedicine, University of Bergen, Bergen, Norway.; ^3^ Department of Medicine, Haukeland University Hospital, Bergen, Norway.

## Abstract

Autoimmune Addison's disease (AAD) is a disorder caused by an immunological attack on the adrenal cortex. The interferon (IFN)-inducible chemokine CXCL10 is elevated in serum of AAD patients, suggesting a peripheral IFN signature. However, CXCL10 can also be induced in adrenocortical cells stimulated with IFNs, cytokines, or microbial components. We therefore investigated whether peripheral blood mononuclear cells (PBMCs) from AAD patients display an enhanced propensity to produce CXCL10 and the related chemokine CXCL9, after stimulation with type I or II IFNs or the IFN inducer poly (I:C). Although serum levels of CXCL10 and CXCL9 were significantly elevated in patients compared with controls, IFN stimulated patient PBMC produced significantly less CXCL10/CXCL9 than control PBMC. Low CXCL10 production was not significantly associated with medication, disease duration, or comorbidities, but the low production of poly (I:C)-induced CXCL10 among patients was associated with an AAD risk allele in the phosphatase nonreceptor type 22 (*PTPN22*) gene. PBMC levels of total STAT1 and -2, and IFN-induced phosphorylated STAT1 and -2, were not significantly different between patients and controls. We conclude that PBMC from patients with AAD are deficient in their response to IFNs, and that the adrenal cortex itself may be responsible for the increased serum levels of CXCL10.

## Introduction

Many patients with autoimmune diseases have signs of a continuous production of type I interferons (IFNs) and display an increased expression of IFN-regulated genes (Ronnblom 2011). In particular, patients with systemic lupus erythematosus (SLE) have increased serum activity of IFN-α and excessive signatures of interferon-stimulated genes (ISGs) in peripheral blood leukocytes (Blanco and others 2001; Bennett and others 2003). Clinical treatment of infectious or malignant disorders with type I IFNs have also been shown to induce autoantibodies and overt autoimmune disease, hence, indicating a role for IFNs in breaking tolerance and promote on-going autoimmune reactions in man (Karlsson-Parra and others 1990). In particular, endocrine glands appear susceptible to IFN-induced autoimmune disease (Malik and others 2001; Michels and Eisenbarth 2010). Prospective studies have shown that up to 15% of patients receiving pegylated IFN-α therapy for chronic hepatitis C virus (HCV) infections develop clinical thyroiditis, while 40% develop thyroid autoantibodies (Tomer 2010). Moreover, development of type 1 diabetes (T1D) during or shortly after IFN-α therapy has been reported (Nakamura and others 2011). The involvement of the type I IFN pathway have been suggested for both thyroiditis and T1D by increased serum activity of type I IFNs (Mavragani and others 2013). The principal type II IFN, IFN-γ, is also heavily implicated in these disorders as one of the major cytokines produced by antigen-specific autoreactive T cells (Weetman 2004; Arif and others 2014).

One archetypical ISG is the proinflammatory chemokine CXCL10, which is strongly upregulated in response to both type I IFNs and IFN-γ (Groom and Luster 2011). CXCR3, the receptor for CXCL10, is highly expressed on activated T cells implicating CXCL10 as an important mediator in the recruitment of T cells to sites of inflammation (Qin and others 1998). Elevated serum levels of this particular chemokine have been demonstrated in many autoimmune diseases, including autoimmune thyroiditis and T1D (Antonelli and others 2014) and in autoimmune Addison's disease (AAD) (Rotondi and others 2005; Kisand and others 2008; Bellastella and others 2011; Bratland and others 2013; Ekman and others 2014). AAD is a classic organ-specific endocrine autoimmune disease, characterized by highly targeted immune responses against specific antigens in the adrenal cortex, in particular cytochrome P450 steroid 21-hydroxylase (21OH) (Bratland and Husebye 2011). The development of AAD is believed to be multifactorial, with several predisposing genetic factors (Mitchell and Pearce 2012). Among these is the C1858T allele in the lymphoid tyrosine phosphatase nonreceptor type 22 (*PTPN22*) gene, a susceptibility allele for numerous autoimmune diseases (Skinningsrud and others 2008; Roycroft and others 2009). This single nucleotide polymorphism (SNP) causes an amino acid shift from arginine to tryptophan at position 620, affecting the functional properties of PTPN22, including its recently described role as a regulator of IFN production in myeloid cells (Bottini and others 2004; Wang and others 2013).

In addition to genetic and environmental factors, the cells of the adrenal cortex probably also play an important role in the pathogenesis of AAD. In this context it has been suggested, and also experimentally demonstrated, that adrenocortical cells subjected to IFNs or cytokines are able to attract 21OH-specific autoreactive T cells in a CXCL10-specific manner (Kisand and others 2008; Bratland and others 2013; Hellesen and others 2014). However, although adrenocortical cells have been shown to produce large amounts of CXCL10 upon IFN stimulation (Rotondi and others 2005; Bratland and others 2013); it is still possible that the elevated CXCL10 serum levels in AAD patients are at least partially produced peripherally by leukocytes (T cells or monocytes) due to an active IFN signature in the peripheral blood. In this study we therefore characterized the peripheral blood immune cell response to IFNs in AAD patients, in particular with regard to CXCL10 production. The data presented offer increased insight into the pathogenesis of AAD, which is essential for future development of novel therapeutic strategies complementing simple hormone replacement therapy.

## Materials and Methods

### Patients and controls

Through the Norwegian registry and biobank for organ-specific autoimmune diseases we have access to serum, plasma, and peripheral blood mononuclear cells (PBMCs) from patients with confirmed AAD. In total, 53 consecutively selected patients were recruited for this study. Seventy-five age- and gender-matched healthy controls were recruited from the local blood bank and from volunteers. Samples from all patients and controls were not available for all experiments (exact numbers are addressed in the figure legends). All patients and controls signed informed consent approved by the Health Region West Ethics Committee (149/96-47.96) and all experiments were conducted in accordance with the Declaration of Helsinki.

### PBMC isolation and stimulation

Heparinized blood samples from 19 AAD patients (see Table 1 for patient details) and 21 controls were processed essentially as described previously (Bratland and others 2013). In brief, plasma samples were isolated, aliquoted, and kept frozen at −20°C, while PBMC were isolated using Ficoll-Paque Plus (GE Healthcare). Upon isolation PBMC were kept cryopreserved at −150°C in 90% AB serum (Lonza) and 10% dimethylsulphoxide (DMSO). PBMC stored at −150°C were thawed, washed, and resuspended in serum-free AIM V medium (Life-technologies). The cells were seeded at 1×10^6^ cells in 500 μL medium in 24-well culture plates, and stimulated with various IFNs or polyinosinic:polycytidylic acid (poly (I:C)) depending on the total number of cells available: IFN-α (PBL Biomedical laboratories) at 10^4^ U/mL, IFN-β (RnD Systems) at 10^4^ U/mL, IFN-γ (Biolegend) at 1 μg/mL, and poly (I:C), (Invivogen) at 10 μg/mL. Optimal doses for each stimulus were determined in preliminary experiments. Nonstimulated cells grown in medium alone served as controls. The concentrations of chemokines or IFNs induced by medium alone were subtracted from the IFN- or poly (I:C)-induced levels. The cells were grown for 24 h at 37°C with 5% CO_2_ in a humidified incubator, upon which cells and supernatants were harvested and stored at −80°C for RNA isolation and downstream assays, respectively.

**Table 1.** Information About the Patients Used in the Stimulation Experiments

*Patient no.*	*Sex*	*Disease duration*	*Age*	*Comorbidity*	*Corticosteroid rates (mg/day)*	*PTPN22 1858 genotype*
1	F	19	46	—	25	C-C
2	F	31	55	—	30	C-C
3	M	15	41	T1D, V, Gr	37.5	C-C
4	F	0	26	T1D	n/a	C-C
5	M	15	55	—	31.25	C-C
6	F	14	58	—	37.5	C-C
7	F	12	49	—	37.5	C-C
8	F	9	61	HT	25	C-C
9	F	11	24	—	32	C-C
10	F	6	35	HT	37.5	C-C
11	F	1	52	Al	25	C-C
12	F	1	26	—	n/a	C-C
13	M	2	26	—	37.5	T-C
14	F	0	21	—	50	T-C
15	F	1	30	T1D, HT	25	T-C
16	M	1	60	—	n/a	T-C
17	F	0	40	HT	37.5	T-C
18	F	60	80	—	31.25	T-T
19	F	0	44	T1D, HT	n/a	T-T

Al, alopecia; Gr, graves; HT, Hashimoto's thyroiditis; n/a, not available; PTPN22, phosphatase nonreceptor type 22; T1D, type 1 diabetes; V, vitiligo.

### ELISA for CXCL9 and CXCL10

Cell supernatants were tested for CXCL9 and CXCL10 content using DuoSet Sandwich ELISA kits from RnD Systems. Plasma and serum levels of CXCL9 and CXCL10 were determined by sandwich ELISA kits validated for plasma/serum analyses from RayBiotech, Inc.

### RNA isolation from cultured PBMCs and cDNA synthesis

Cultured PBMC were lysed in lysis buffer RLT (Qiagen) and stored at −80°C. RNA was extracted using RNeasy Mini Kit, Shredder and RNase Free Dnase Set (all from Qiagen), and then purified and concentrated using RNeasy MinElute Cleanup Kit (Qiagen) according to the manufacturer's protocol. Purified RNA was stored at −80°C before total RNA was reverse transcribed using iScript cDNA synthesis Kit (Biorad), according to manufacturer's protocol.

### Quantitative real-time polymerase chain reaction

Primers were designed using Primer-BLAST (NCBI), supplied by Life Technologies/Invitrogen and verified by standard curve assessment (Table 2). Real-time polymerase chain reaction (PCR) was performed in 10 μL sample volume using Roche LightCycler 480 system containing 5 μL iQ Sybr Green Supermix (BioRad Laboratories Ltd), 0.5 μL of each primer at 10 nM and 4 μL of the previous reverse-transcribed cDNA template. Two endogenous controls, porphobilinogen deaminase (PBGD) and hypoxanthine phosphoribosyl transferase 1 (HPRT1) were used for normalization. The protocol used is as follows: denaturation (95°C for 5 min), amplification repeated 40 times (95°C for 10 s, 60°C for 15 s, and 72°C for 15 s). A melt curve analysis (95°C for 5 s, 65°C for 1 min, and then heated to 97°C at 0.11°C/s) was performed following every run to ensure a single amplified product for every reaction. All reactions were carried out in triplicate and repeated 2 or 3 times along with 2 negative controls (cDNA synthesized RNA without reverse transcriptase and no template cDNA).

**Table 2.** Primers Used for Quantitative Real-Time Polymerase Chain Reaction

*Gene*	*Primer sequence*
*USP18*	FP: 5′-CGTGGAACTCAGCAGCGG-3′
	RP: 5′-TCAGGACAGCACGACTTCACTT-3′
*EIF2AK2*	FP: 5′-TCGCAAGACTATGGAAAGGAAG-3′
	RP: 5′-CATCCCGTAGGTCTGTGAAAAA-3′
*CYP2E1*	FP: 5′-GACCTGTTCTTTGCGGGGA-3′
	RP: 5′-CTTGATGGCAGGGATTCGG-3′
*CXCL10*	FP: 5′-GAACCTCCAGTCTCAGCACC-3′
	RP: 5′-TGCAGGTACAGCGTACAGTT-3′
*IRF7*	FP: 5′-GAGCTGTGCTGGCGAGAAG-3′
	RP: 5′-GGAGTCCAGCATGTGTGTGT-3′
*PBGD*	FP: 5′-GAGCCAAGGACCAGGACATCT-3′
	RP: 5′-AGTCAGGTACAGTTGCCCATCC-3′
*HPRT1*	FP: 5′-GCTTTCCTTGGTCAGGCAGTA-3′
	RP: 5′-AACACTTCGTGGGGTCCTTT-3′

### Bioassay for type I IFNs

The biological activity of type I IFNs in cell culture supernatants were estimated using a commercial reporter cell-line, HEK Blue IFN-α/β (Invivogen), which is stably transfected with the necessary components of a fully functional type I IFN pathway and a reporter gene expressing a secreted embryonic alkaline phosphatase (SEAP) under control of the *ISG54* gene. HEK Blue IFN-α/β cells were maintained in DMEM medium with Glutamax and high glucose (4.5 g/L), supplemented with 10% (v/v) fetal bovine serum, 50 U/mL streptomycin, 100 μg/mL normocin, 30 μg/mL blasticidin, and 100 μg/mL Zeocin. Cells were cultured at 37°C, 5% CO_2_, passaged at 70%–80% confluency and maintained for no longer than 3 weeks to ensure genetic stability. To estimate IFN activity, 20 μL of cell culture supernatants were added to flat-bottomed 96-well culture plates along with 5×10^4^ HEK Blue IFN-α/β cells. Neutralizing monoclonal antibodies against IFN-β (clone MMHB-2, PBL Assay Science) were added to selected wells (10 μg/mL) to estimate the relative amounts of each type I IFN (-α or -β) produced. A standard curve was also prepared using 2 fold dilutions of IFN-α over the range of 1.56–100 U/mL. After 20–24 h of cell culture 20 μL of the induced HEK Blue IFN-α/β supernatant were mixed with 180 μL QUANTI-Blue (Invivogen) SEAP substrate, a medium that turns blue/purple in the presence of SEAP. After 2 h of incubation at 37°C, the absorbance was measured at 650 nm, corresponding to the amount of SEAP produced in response to IFNs.

### Analysis of ISG expression

For the analysis of basic gene expression of selected IFN-stimulated genes, peripheral blood was collected from 15 AAD patients and 15 healthy controls and CD4^+^ T cells were isolated by the Dynabeads CD4 positive Isolation Kit (Life Technologies/Invitrogen) according to the manufacturer's protocol. Total RNA was immediately extracted using RNeasy Mini Kits (Qiagen) following the manufacturer's protocol and DNase I (Qiagen) was added to remove remaining genomic DNA. Samples were stored at −80°C before 1 μg of total RNA was reverse transcribed using iScript cDNA synthesis Kit (Biorad) in total volume of 40 μL, according to manufacturer's protocol. For the identification of an IFN signature, we used a set of 5 genes previously identified as IFN-regulated in peripheral blood after short-term treatment with both type I and II IFNs: *CXCL10*, *CYP2E1*, *EIF2AK2*, *IRF7*, and *USP18*. All selected IFN-stimulated genes were previously shown to contain differentially methylated regions in AAD patients compared with healthy controls (Bjanesoy and others 2014). Quantitative real-time PCR was performed as described above. Relative quantification of the gene expression levels was calculated for each individual by comparison with an average of all Ct values measured in healthy blood donors and by normalization against the reference genes *PBGD* and *HPRT1*. Individual IFN scores were then calculated essentially as described previously (Bilgic and others 2009): The 95th percentile of expression levels was calculated for each gene, and all measured individual expression levels were normalized to the calculated 95th percentile value. Expression values equal to or greater than the 95th percentile were replaced with the 95th percentile so that the maximum value for each gene was 1.0. The normalized expression levels for the 5 analyzed genes were then summarized to yield the final IFN score.

### Analysis of STAT1 and STAT2 activation

To investigate if there were any differences in the STAT1/2 activation of stimulated cells in patients and controls, total and phosphorylated STAT levels were measured using cell-based ELISA immunoassays for human phospho-STAT 1 and 2 from R&D systems. The assay procedure from the manufacturer was followed, using the protocol for nonadherent cells. The cells were seeded at a concentration of 1.0×10^6^ cells/mL and stimulated for 15 min with the same concentration of stimuli (IFNs-α, -β, -γ and poly (I:C)) used in the PBMC stimulation experiments.

### Genotyping of *PTPN22* SNP

For all patients who donated cells to stimulation experiments (*n*=19), genomic DNA was extracted from peripheral whole blood using QIAamp DNA Blood Mini kit (Qiagen). Genotyping was conducted by means of PCR followed by a restriction fragment length polymorphism assay (PCR-RFLP) essentially as described previously (Bottini and others 2004). A restriction site for the enzyme *Xcm*I is present in the PCR amplified fragment when an individual is carrying the 1858T *PTPN22* allele, but not when the person is homozygous for the 1858C *PTPN22* allele, meaning that the *Xcm*I enzyme cleaves the PCR product in the presence of the T nucleotide. Cleavage of the PCR products was visualized with electrophoresis on a 3% agarose gel using GelRed. Genotypes for all patients were also confirmed using conventional Sanger DNA sequencing of ExoProStar (GE Healthcare) cleaned PCR products.

### Statistics

Statistical differences between patients and controls or between different patient groups were calculated using nonparametric Mann–Whitney *U*-test. For correlation analyses, a nonparametric Spearman test was used. All quantitative data are expressed as the mean of duplicates or triplicates. For all statistical operations, 2-tailed tests were used and *P*<0.05 was considered significant. All tests were performed with GraphPad Prism v5.02.

## Results

### Increased serum levels of CXCL9 and CXCL10 in AAD patients

**FIG. 1.**
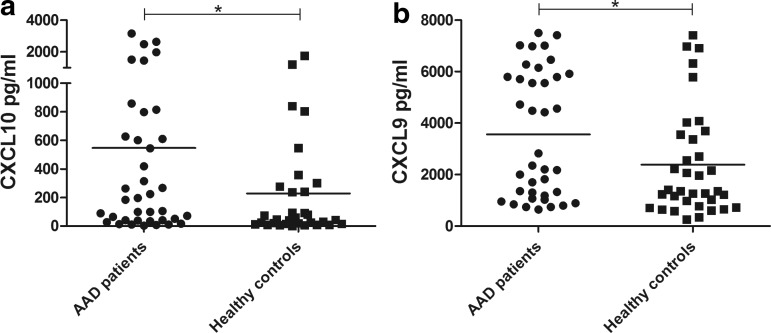
Plasma/serum levels of CXCL10 and CXCL9. Sandwich ELISA was used to assay for CXCL10 **(a)**, and CXCL9 **(b)** contents in serum/plasma samples from AAD patients (*n*=38) and healthy controls (*n*=35 for CXCL10, *n*=32 for CXCL9). The results for each patient and control are displayed as means of duplicates and the bars display the mean for the whole group. Nonparametric Mann–Whitney *U*-test was used to test for statistical differences between patients and controls (*P<0.05). AAD, autoimmune Addison's disease.

Previous reports have shown that CXCL10 levels are elevated in AAD patients (Rotondi and others 2005; Bratland and others 2013), so we initially determined the serum levels of CXCL10 and CXCL9 in our patient cohort. We observed a significant difference in the CXCL10 levels between patients and controls, where the mean was 546 pg/mL in patients (range 3.4–3,153 pg/mL) and 228 pg/mL in controls (range 0.0–1,757 pg/mL) (*P*<0.05, Fig. 1a). A significant difference was also observed for the serum levels of CXCL9 (AAD patients, mean 3,561 pg/mL, range 644–7,500 pg/mL; controls, mean 2,384 pg/mL, range 259–7,419 pg/mL) (*P*<0.05, Fig. 1b). There were no significant differences in serum levels of CXCL10 or CXCL9 between patients with isolated AAD and patients with autoimmune polyendocrine syndrome type 2 (APS-2), defined as AAD plus additional autoimmune endocrinopathies (Supplementary Fig. S1; Supplementary Data are available online at www.liebertpub.com/jir).

### Deficient chemokine production in IFN-stimulated PBMC from AAD patients

**FIG. 2.**
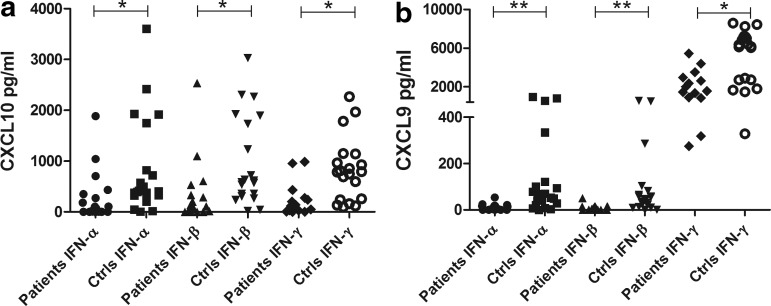
Levels of CXCL10 and CXCL9 production in PBMC stimulated with type I and II interferons (IFNs). Sandwich ELISA was used to assay for CXCL10 **(a)** and CXCL9 **(b)** contents in supernatants from cells stimulated with type I (IFN-α and IFN-β at 10^4^ U/mL) and type II (IFN-γ at 1 μg/mL) IFNs for 24 h. The results for each patient (IFN-α, *n*=19; IFN-β, *n*=18; IFN-γ, *n*=14) and control (IFN-α, *n*=21; IFN-β, *n*=20; IFN-γ, *n*=20) are displayed as means of duplicates. Nonparametric Mann–Whitney *U*-test was used to test for statistical differences between patients and controls for each IFN (*P<0.05, **P<0.001). PBMC, peripheral blood mononuclear cell.

**FIG. 3.**
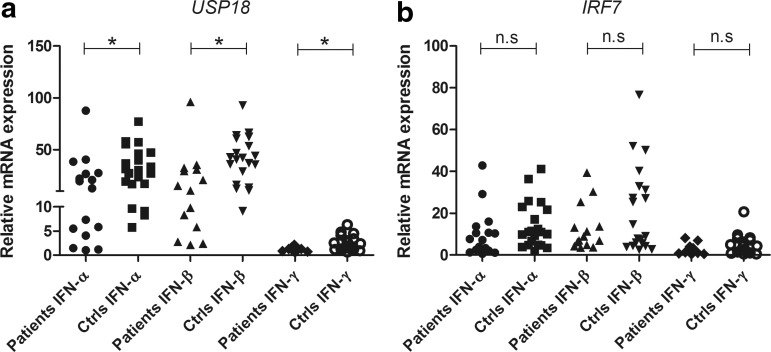
Relative mRNA expression of *USP18* and *IRF7* in PBMC stimulated with type I and II IFNs. Real-time qPCR was used to determine the relative mRNA expression of *USP18* **(a)** and *IRF7* **(b)** in PBMC stimulated with type I (IFN-α and IFN-β at 10^4^ U/mL) and type II (IFN-γ at 1 μg/mL) IFNs for 24 h. The results for each patient (IFN-α, *n*=19; IFN-β, *n*=18; IFN-γ, *n*=14) and control (IFN-α, *n*=21; IFN-β, *n*=20; IFN-γ, *n*=20) are displayed as means of triplicates. Nonparametric Mann–Whitney *U*-test was used to test for statistical differences between patients and controls for each IFN (*P<0.05). n.s., not significant; qPCR, quantitative real-time polymerase chain reaction.

To investigate whether the increased serum levels of CXCL10 in AAD patients reflect an increased IFN activity in the patients' peripheral blood, we stimulated PBMC from patients and controls with types I and II IFNs and determined the production of CXCL10. The levels of CXCL10 were significantly lower in AAD patients than controls, regardless of the source of IFN stimuli (*P*<0.05 for IFN-α, -β and -γ, Fig. 2a). We also assessed the production of CXCL9, a chemokine preferentially induced by IFN-γ and only by a lesser degree by type I IFNs. As for CXCL10, the induction of CXCL9 in PBMC was significantly impaired in AAD patients compared with controls for both type I and II stimulation (*P*<0.001 for IFN-α and -β, *P*<0.05 for IFN-γ, Fig. 2b). In a subset of patients and controls, we also measured the levels of IFN-γ and TNF-α produced after stimulation with IFN-α and -β (Supplementary Fig. S2). Both cytokines were detectable at rather low levels in both patients and controls. No significant differences were noted. Resting of the cells for 24 h before stimulation did not appear to improve the CXCL10 production from the patients' PBMC relative to that of the controls. Patient cells that had rested for 24 h before stimulation with IFN-α showed both higher and lower production of CXCL10 than cells stimulated directly (Supplementary Fig. S3), while all the control cells tested had slightly increased CXCL10 production in cells that were stimulated after 24 h of resting. To assess whether the deficient response to IFNs extended to other parts of the IFN pathways we determined the relative mRNA levels of the 2 ISGs, *USP18*, and *IRF7* in response to IFN stimulation of PBMC (Fig. 3). The mRNA levels of USP18 were significantly lower in patients compared with controls for all 3 IFNs (*P*<0.05 for IFN-α, -β, and -γ, Fig. 3a). For IRF7 no significant difference in expression level was observed, although the mean values were higher for controls for all IFNs (Fig. 3b).

### Deficient chemokine and IFN production in poly (I:C)-stimulated PBMC from AAD patients

**FIG. 4.**
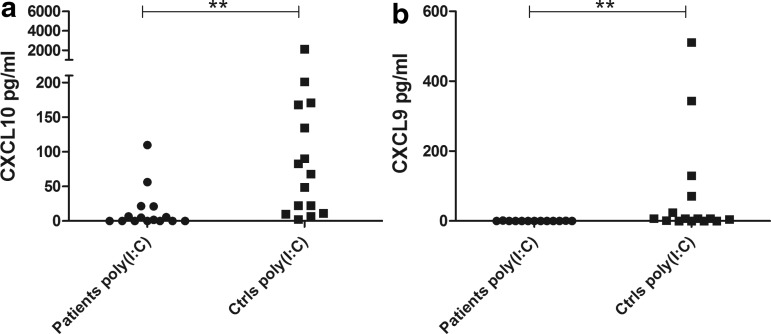
Levels of CXCL10 and CXCL9 production in PBMC stimulated with poly (I:C). Sandwich ELISA was used to assay for CXCL10 **(a)** and CXCL9 **(b)** contents in supernatants from cells stimulated with poly (I:C) (10 μg/mL) for 24 h. The results for each patient (CXCL10, *n*=15; CXCL9, *n*=13) and control (*n*=15) are displayed as means of duplicates. Nonparametric Mann–Whitney *U*-test was used to test for statistical differences between patients and controls (**P<0.001).

**FIG. 5.**
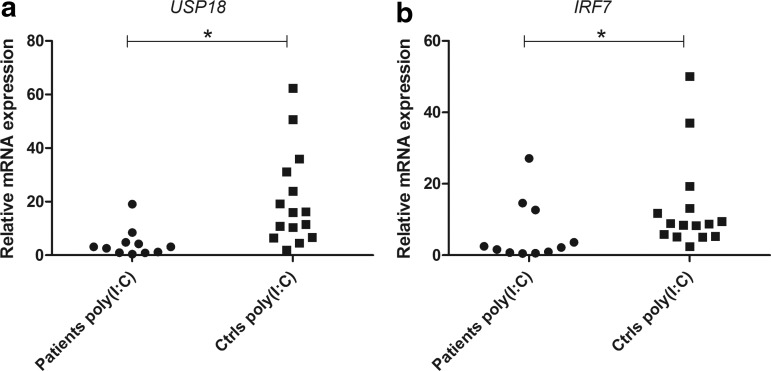
Relative mRNA expression of *USP18* and *IRF7* in PBMC stimulated with poly (I:C). Real-time qPCR was used to determine the relative mRNA expression of *USP18* **(a)** and *IRF7* **(b)** in PBMCs stimulated with poly (I:C) (10 μg/mL) for 24 h. The results for each patient (*n*=11) and control (*n*=15) are displayed as means of triplicates. Nonparametric Mann–Whitney *U*-test was used to test for statistical differences between patients and controls (*P<0.05).

**FIG. 6.**
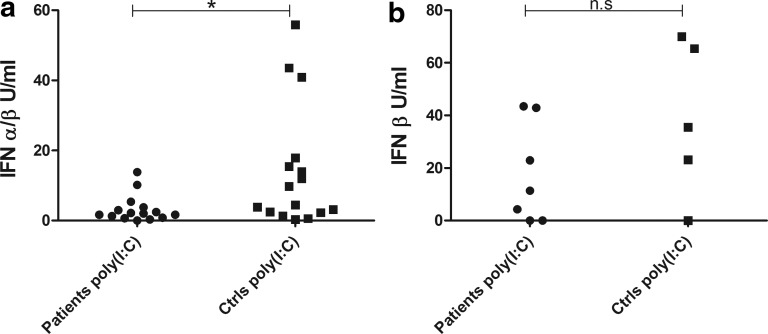
Levels of type I IFNs in PBMC stimulated with poly (I:C). Bioassay for type I IFNs was used to measure content of total type I IFN **(a)** in supernatants from PBMC stimulated with poly (I:C) (10 μg/mL) for 24 h. The results for each patient (*n*=15) and control (*n*=16) are displayed as means of duplicates. IFN-β **(b)** content was estimated using a modified bioassay with neutralizing anti-IFN-β antibodies in 7 patients and 5 controls [using the same supernatants as in **(a)**]. Nonparametric Mann–Whitney *U*-test was used to test for statistical differences between patients and controls (*P<0.05).

Because poly (I:C) is a TLR3 agonist mimicking viral dsRNA and a well-known inducer of type I IFNs (and therefore an indirect inducer of CXCL10), we determined whether PBMC from the patients would respond differently to poly (I:C) stimulation than healthy controls. The levels of both CXCL10 and CXCL9 produced by PBMC were significantly reduced in patients (*P*<0.001, Fig. 4). As for IFN-α, patient cells that were stimulated with poly (I:C) after 24 h of resting did not show consistently increased CXCL10 production compared to cells stimulated directly at the start of culture (data not shown). The mRNA levels of both *USP18* and *IRF7* were also significantly lower in patients than controls after poly (I:C) stimulation (*P*<0.05 for both *USP18* and *IRF7*, Fig. 5). The production of type I IFNs (IFN-α and -β) in PBMC stimulated with poly (I:C) were also investigated with a cellular reporter assay and again found significantly lower in AAD patients compared with healthy controls (*P*<0.05, Fig. 6a). In a subset of samples, using a modified version of the cellular reporter assay, we also estimated the relative production of IFN-β after poly (I:C) stimulation (Fig. 6b). Although the mean production was higher among controls than the patients, the difference was not statistically significant.

### Correlations between chemokine production and AAD patient characteristics

There were no significant differences in IFN- or poly (I:C)-induced CXCL10 production between patients with isolated AAD and APS-2 (Supplementary Fig. S4). For CXCL9, PBMC from APS-2 patients produced significantly higher levels after IFN-α and -β stimulation than PBMC from patients with isolated AAD (Supplementary Fig. S5). Furthermore, for the patients there were no significant correlations between IFN- or poly (I:C)-induced CXCL10 or CXCL9 production and daily dose of cortisone acetate (Supplementary Figs S6 and S7). With regard to disease duration, no significant correlations were found for IFN- or poly (I:C)-induced CXCL10 production, but there were statistically significant inverse correlations between IFN-α and β-induced CXCL9 and disease duration (Supplementary Figs S8 and S9). When considering the age of the patients, a statistically significant inverse correlation was found between IFN-γ-induced CXCL10 and age (Supplementary Fig. S10). No other significant correlations were found between age and IFN- or poly (I:C)-induced CXCL10 or CXCL9 (Supplementary Fig. S11) production.

### No evidence of an activated IFN signature in the peripheral blood of AAD patients

**FIG. 7.**
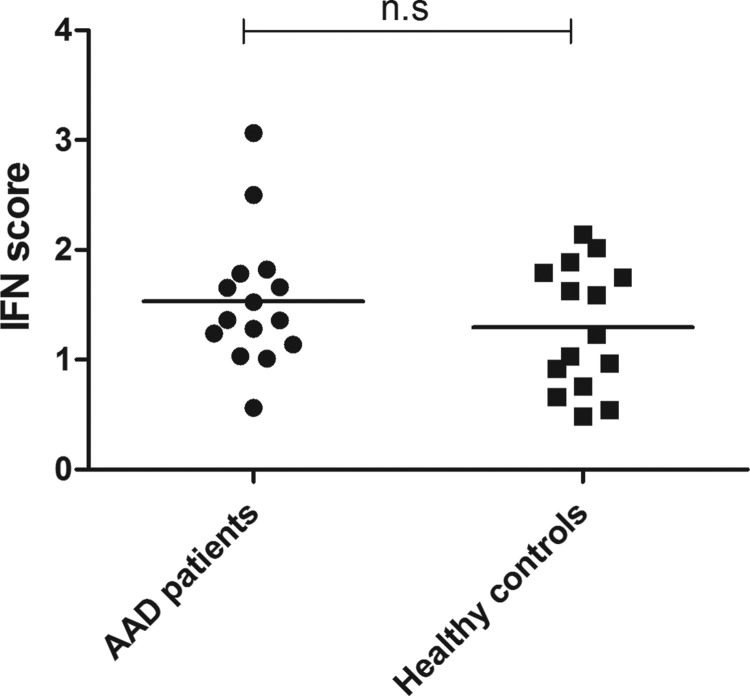
IFN signature genes are not upregulated in the peripheral blood of AAD patients. Real-time qPCR was used to determine the relative mRNA expression of 5 selected ISGs in isolated CD4^+^ T cells. The IFN score for each patient (*n*=15) and control (*n*=15) was calculated as described in the Materials and Methods section. The bars display the mean for the whole group. ISGs, interferon-stimulated genes.

To investigate the potentially dysregulated IFN pathway in the peripheral blood of AAD patients, we compared the expression profiles of 5 selected ISGs in 15 patients with 15 healthy controls. Although some patients clearly showed signs of an activated IFN system, no significant differences could be detected between patients and controls (Fig. 7). Furthermore, no significant differences could be detected for any of the single 5 selected ISGs (Supplementary Fig. S12).

### IFN-stimulated levels of phosphorylated STAT1 and STAT2 are not different in PBMC from AAD patients compared to controls

**FIG. 8.**
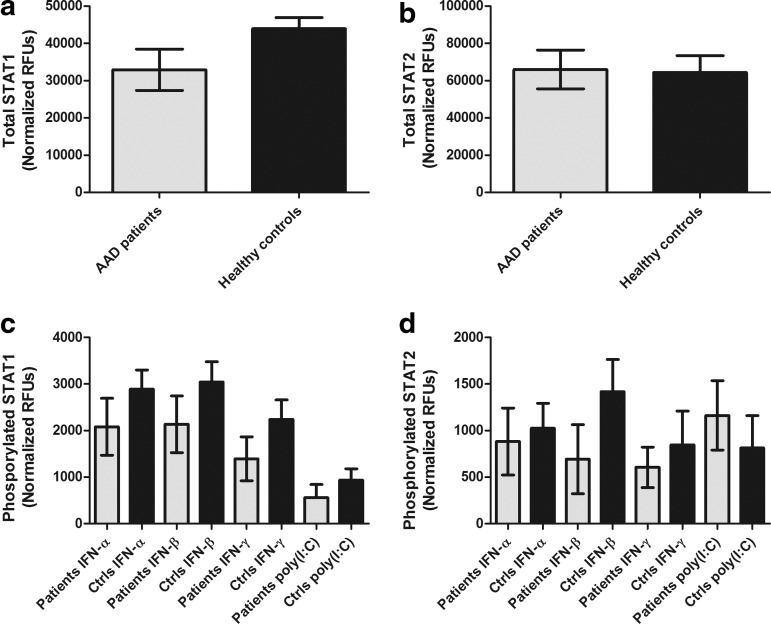
Variations in STAT1/2 and STAT1/2 phosphorylation in cells from patients and controls stimulated with IFNs and poly (I:C). Using a cell-based ELISA PBMC from patients and controls were stimulated for 15 min with IFNs and poly (I:C), using the same concentration as in previous experiments. Total STAT1 **(a)** and total STAT2 **(b)** are illustrated as mean levels for the whole groups with standard error of the mean (SEM). Phosphorylated STAT1 **(c)** and STAT2 **(d)** are shown as group means with SEM for each stimulus in patients and controls. Nonparametric Mann–Whitney *U*-test was used to test for statistical differences between the total STAT1 and -2 and phosphorylated STAT1 and -2 in patients and controls (STAT1, *n*=8; STAT2, *n*=7), but none were found.

We subsequently investigated whether the poor response to IFNs seen in the patients' PBMC could be attributed to deficient expression or phosphorylation of STAT1 and -2 upon IFN stimulation. In unstimulated PBMC no significant differences in total STAT1 or -2 levels were evident between patients and controls (Fig. 8a, b). Furthermore, although mean levels of phosphorylated STAT1 and -2 were consistently lower in IFN- or poly (I:C)-stimulated PBMC from patients compared with controls, no statistically differences were detected (Fig. 8c, d).

### Deficient responses to poly (I:C) associate with *PTPN22* 1858 genotype

**FIG. 9.**
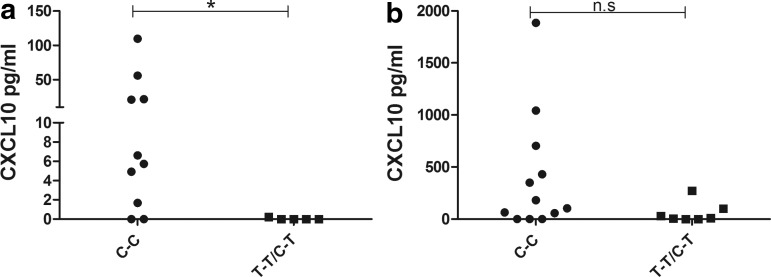
Variations in poly (I:C) and IFN-α-induced CXCL10 production in patients with different *PTPN22* 1858 genotypes. When the poly (I:C)-induced CXCL10 production was stratified by *PTPN22* genotype a significant correlation between carrying the T allele and low production of CXCL10 was found **(a)**. Similar trends were found when IFN-induced CXCL10 production was stratified according to *PTPN22* genotype, although not reaching statistical significance, as shown for IFN-α **(b)**. Nonparametric Mann–Whitney *U*-test was used to test for statistical differences between the different genotypes (*P<0.05). PTPN22, phosphatase nonreceptor type 22.

To find any characteristics among the AAD patients that could help to explain their poor response to IFNs, we looked in the literature and noted that a SNP of the *PTPN22* gene (rs2476601) (which is associated with increased risk for developing AAD) was also associated with impaired IFN response after TLR stimulation in healthy individuals (Wang and others 2013). Hence, all patients that donated PBMCs to the stimulation experiments were genotyped for the *PTPN22* SNP at nucleotide 1858. Only 2 patients were homozygous for the 1858T allele, while 5 were heterozygous carriers. When stimulated with poly (I:C), there was a significant difference in the production of CXCL10 between carriers of the 1858T allele and the 1858C homozygotes (*P*<0.05, Fig. 9a). When the patients carrying the T allele were removed from the original statistical calculations, the difference in the poly (I:C)-induced production of CXCL10 was still significant, but the level of significance dropped (*P* value with T carriers=0.001, *P* value without T carriers=0.0096). Although no other statistically significant associations were found, mean CXCL10 levels were lower in T carriers also after IFN stimulation (as shown for IFN-α, Fig. 9b and IFN-β and -γ, Supplementary Fig. S13). CXCL9 levels after IFN- or poly (I:C) stimulation on the other hand seemed completely independent of *PTPN22* genotype (Supplementary Fig. S14).

## Discussion

This study was performed to investigate whether an abnormal IFN signature is present in the peripheral blood of patients with AAD, and if this can explain the elevated serum levels of CXCL10 in AAD patients. The involvement of IFNs, either type I or II or both, in the immunopathogenesis of AAD is strongly suggested by several observations in both clinical and experimental settings: Individuals treated with IFN-α for viral infections or cancer have developed autoantibodies against 21OH, with or without concomitant clinical adrenocortical insufficiency (Wesche and others 2001; Tran and others 2008; Krysiak and others 2011). Individuals with subclinical and established AAD treated with IFN-α have shown an exacerbation of the condition and an increased need for glucocorticoid replacement (Knost and others 1981; Oshimoto and others 1994). Furthermore, we and others have shown experimentally that types I and III IFNs are directly cytotoxic to adrenocortical cells (van Koetsveld and others 2006; Hellesen and others 2014). Autoreactive 21OH-specific T cells producing large amounts of IFN-γ are also frequent in AAD patients (Bratland and others 2009; Rottembourg and others 2010; Dawoodji and others 2014), and tissue from deceased AAD patients have revealed high expression of HLA class II on adrenocortical cells, suggesting that these cells were indeed exposed to IFN-γ *in vivo* (Jackson and others 1988).

Serum levels of CXCL10 and the related CXCL9 were significantly elevated in the AAD patients recruited to this study. There were no associations between serum chemokine levels and disease duration or comorbidities. It has previously been hypothesized that the elevated CXCL10 levels in serum are produced locally in the adrenal cortex due to the presence of high levels of IFNs (Rotondi and others 2005; Kisand and others 2008; Bratland and Husebye 2011; Bratland and others 2013). The source of these IFNs could either be IFN-γ secreting autoreactive T cells in the process of killing adrenocortical cells, or an ongoing viral infection inducing type I or III IFNs locally (by adrenocortical cells themselves and/or by resident tissue macrophages). However, CXCL10 in serum could also be produced by peripheral blood cells (lymphocytes or monocytes) due to an ongoing inflammatory process or an active IFN signature in the AAD patients. Both excessive and deficient CXCL10 production by PBMC upon IFN stimulation has been described for other autoimmune disorders with a defined peripheral IFN signature, such as SLE and rheumatoid arthritis (Karonitsch and others 2009, 2012).

For all IFN subtypes tested, both CXCL10 and CXCL9 production by PBMCs were significantly lower in AAD patients than controls. For some patients, no chemokine production could be detected at all, despite the relatively high doses of IFNs used for stimulation. Furthermore, there were no obvious associations between chemokine levels and disease duration or comorbidities of the patients, or between the serum chemokine levels and the amount produced by PBMCs after IFN stimulation. As expected, while CXCL10 was induced at equal degrees by all IFNs, CXCL9 was predominantly induced by IFN-γ (Groom and Luster 2011). The small amount of CXCL9 induced by IFN-α/β stimulation could in fact be a result of IFN-γ, either alone or in synergy with TNF-α, induced by the type I IFNs (Sareneva and others 1998). IFN-γ and TNF-α production was indeed detectable in cell supernatants after type I IFN stimulations. No significant differences were noted between patients and controls for the type I IFN induced IFN-γ and TNF-α, but as the number of individuals tested was small this should be interpreted with caution. There were significant negative correlations between IFN-α and β-induced CXCL9 and disease duration, which could indicate that the IFN-γ production in AAD patients with long lasting disease is deficient and that this might partially explain the low CXCL9 production. The fact that both CXCL10 and CXCL9 production was decreased in the patients indicated that both type I and II IFN signaling pathways were deficient.

To further delineate the cause of the decreased chemokine production the expression of 2 essential ISGs in the IFN-stimulated PBMC, *USP18*, and *IRF7* was investigated. Ubiquitin-specific peptidase 18 (encoded by *USP18*) is a classical ISG that provides a strong negative feedback signal serving to downregulate the activation of the IFN-activated Janus kinase-signal transducer and activator of transcription (Jak-STAT) pathways (Malakhova and others 2006). Importantly, the mRNA expression of *USP18* was significantly lower in AAD patients than healthy controls after IFN stimulation, indicating that the poor response to IFNs is not selective for chemokine production. Interferon regulatory factor 7 (encoded by *IRF7*) is, as opposed to *USP18*, induced in a positive regulatory feedback by type I IFNs during antiviral responses (Marie and others 1998). Viral infections or TLR (e.g. TLR3) engagement activates IRF7, which initially participates in the transcriptional induction of small amounts of type I IFNs. These IFNs then bind to IFN receptors on adjacent cells, which in turn amplifies the activation of IRF7 and consequently also the production of IFNs (Ning and others 2011). However, the mRNA expression of *IRF7* was not statistically different between patients and controls after IFN stimulation, indicating that the deficient chemokine response to direct stimulation with IFNs in AAD patients is independent of IRF7.

To investigate chemokine production elicited by endogenous IFNs, we stimulated patient and control PBMCs with the TLR3 agonist poly (I:C), a well-characterized inducer of IFNs (Alexopoulou and others 2001). The chemokine production was still significantly lower in AAD patients than the controls. We also assessed the endogenous type I IFN production itself, since the low production of CXCL10 after poly (I:C) stimulation could either reflect the poor response to direct stimulation with IFNs or point to an additional defect in the endogenous production of type I IFNs. Again, there was a statistically significant lower production of type I IFNs in the AAD patients compared with the controls. We also estimated the relative levels of IFN-β induced by poly (I:C), but these levels were not significantly different between patients and controls. Furthermore, the expression levels of *USP18* were significantly lower among AAD patients than healthy controls, probably reflecting the decreased levels of poly (I:C)-induced type I IFN in the patients. In contrast to the findings with exogenous IFNs, the poly (I:C)-induced gene expression levels of *IRF7* were significantly lower among AAD patients than controls. This could indicate that the low chemokine production in AAD patients is a reflection of poor IFN production, and not primarily a consequence of low sensitivity to exogenous IFNs. This is also supported by the well-known fact that IFN-induced production of ISGs in PBMCs includes a positive feedback loop where additional endogenous types I and II IFNs are induced and participates in enhancing the production of chemokines (Taniguchi and Takaoka 2002).

The poor production of IFN and IFN-induced chemokines in AAD patients could be caused by IFN-induced refractoriness, known from chronic HCV infections where a prolonged endogenous stimulation with type I IFNs induces a desensitization of cells to the IFN pathways, preventing them from responding to exogenous IFNs (Makowska and others 2011). IFN-induced refractoriness is also well described in cell culture and animal models (Larner and others 1986; Sarasin-Filipowicz and others 2009). We therefore looked at the gene expression of 5 classical ISGs in isolated CD4^+^ T cells to investigate whether there were any signs of prolonged activation of the endogenous IFN system in the AAD patients. The 5 chosen ISGs were selected based on a recent genome-wide DNA methylation study showing that they all contained hypomethylated regions in AAD patients compared with healthy controls (Bjanesoy and others 2014). Although a few patients clearly had high expression levels of single ISGs, no statistical difference in the calculated IFN score could be detected between patients and controls. IFN-induced refractoriness is therefore not a likely explanation for the IFN-induced chemokine production in the AAD patients. This is also supported by the fact that even *USP18* were expressed at lower levels in patients than controls after IFN stimulation, given that *USP18* appears to be a key mediator of IFN-induced refractoriness (Sarasin-Filipowicz and others 2009; Francois-Newton and others 2011).

To narrow down our search for possible defects in the molecular pathways of IFN signaling, we assessed the total levels of STAT1 and STAT2 in unstimulated PBMC from AAD patients and controls. We also compared phosphorylated STAT1/STAT2 levels after stimulation with IFNs-α, -β, -γ, or poly (I:C). No significant differences between patients and controls were found for total STAT1/STAT2 levels, or for any IFN or poly (I:C)-stimulated STAT1/STAT2 phosphorylation. These observations indicate that the deficient responses to IFNs and poly (I:C) in AAD patients does not occur at type I or II IFN receptor/TLR3 levels or upstream of STAT signaling. Instead, the defects in the AAD patients may originate from signaling or transcriptional events downstream of STAT1/STAT2 phosphorylation, or at the production level of IFNs.

Another reason for the poor response to and production of IFNs could be that patients with AAD are genetically prone to this phenotype. A recent report showed that PTPN22, traditionally regarded as a regulator of T-cell receptor signaling, is also a regulator of the type I IFN system after TLR stimulation (Wang and others 2013). Since the 1858T allele of the *PTPN22* gene is a risk variant for AAD, we genotyped all the AAD patients that were included in the PBMC stimulation experiments and stratified their genotypes to the their poly (I:C)-induced chemokine production. Strikingly, the patients carrying the 1858T allele appeared to produce less CXCL10 in response to poly (I:C) than patients homozygous for the 1858C allele. The statistically significant difference in CXCL10 production after poly (I:C) stimulation indicates that carriers of the 1858T allele are genetically prone to produce less type I IFN (and hence CXCL10) upon TLR3 stimulation than 1858C homozygotes, consistent with the recently described role for PTPN22 in IFN induction (Wang and others 2013). However, the deficient chemokine response to exogenous IFNs can hardly be explained by this finding, as the IFN regulating role of PTPN22 is upstream of IFN induction.

The decreased production of IFNs and chemokines in AAD could in theory also be related to the cortisol supplementation therapy given to the patients. High-dose intravenous steroid treatment with methylprednisolone efficiently downregulates the IFN signature in SLE leukocytes, although more than 500-fold higher than the normal replacement regimens that AAD patients adhere to are required (Bennett and others 2003; Oksnes and others 2014). Still, it is a well-known fact that glucocorticoids may interfere on the action of IFN pathways in several ways, for example, by inhibiting STAT1 phosphorylation or the heterotrimeric STAT1-STAT2-IRF9 (ISGF3) transcription complex (Hu and others 2003; Bhattacharyya and others 2011). Thus, we performed an experiment where in addition to stimulating the cells with IFNs and poly (I:C) directly, we also rested the cells for 24 h before stimulation. However, no improvement in CXCL10 production from patient PBMC relative to healthy controls was observed. It should also be stressed that patients with AAD have no endogenous glucocorticoid production, and in general AAD patients on supplementation therapy do not have increased serum cortisol levels (Methlie and others 2013). Still, there is a general concern that AAD patients receive more glucocorticoids than the normal endogenous production (Lovas and others 2009; Husebye and others 2014). Notably, however, there were no correlation between the individual patients' daily cortisol replacement doses and chemokine responses to IFN stimulation in this study. This could indicate that the cortisol treatment is not the explanation for the generally decreased response.

There are some limitations to this study that needs to be addressed. First, we generally used total PBMC for all stimulation experiments without taking into consideration the inter-individual variations in the distributions of the PBMC subsets. In particular, the amount of monocytes in a given individual could influence the results. However, we are not aware of any studies reporting that AAD patients have less monocytes among their PBMC than healthy individuals. Furthermore, patients were recruited to this study in a consecutive manner, with no regard to disease duration. Although there were no correlations between disease duration and IFN-stimulated chemokine production, disease duration could be highly relevant when considering the presence of an IFN signature in AAD patients. A recent study of children genetically predisposed to develop T1D revealed the presence of a transient IFN signature before the onset of autoimmune disease (Ferreira and others 2014). Also, it would have been interesting to add a more comprehensive list of ISGs for the studies of a possible IFN signature and for the gene expression studies of IFN- and poly (I:C)-stimulated PBMC. Unfortunately, this was not possible for the current investigation due to a limited number of cells and RNA from the patients.

To conclude, we have demonstrated that despite elevated serum levels of the IFN-induced chemokines CXCL10 and CXCL9, peripheral blood cells from patients with AAD are deficient in the production of these chemokines and there is no evidence for an IFN signature in the peripheral blood. This deficiency may in part be related to a defect in the endogenous production of type I IFNs in peripheral blood cells that again could be linked to an AAD risk allele in the *PTPN22* gene. Taken together, these observations suggest that the elevated CXCL10 levels in the serum of AAD patients instead are locally produced in the adrenal cortex, under the influence of an IFN-rich milieu. Future work should focus on discovering the source and exact identity of these IFNs.

## Supplementary Material

Supplemental data

Supplemental data

Supplemental data

Supplemental data

Supplemental data

Supplemental data

Supplemental data

Supplemental data

Supplemental data

Supplemental data

Supplemental data

Supplemental data

Supplemental data

Supplemental data
