# Supplementary material for: Peripheral Blood Cells from Patients with Autoimmune Addison's Disease Poorly Respond to Interferons In Vitro, Despite Elevated Serum Levels of Interferon-Inducible Chemokines
Source: J Interferon Cytokine Res. 2015 Oct 1;35(10):759–70. doi: 10.1089/jir.2014.0171 (PMC4589105; doi:10.1089/jir.2014.0171)
Supplement: Supplemental data [file Supp_Fig1.pdf]

Supplementary Data

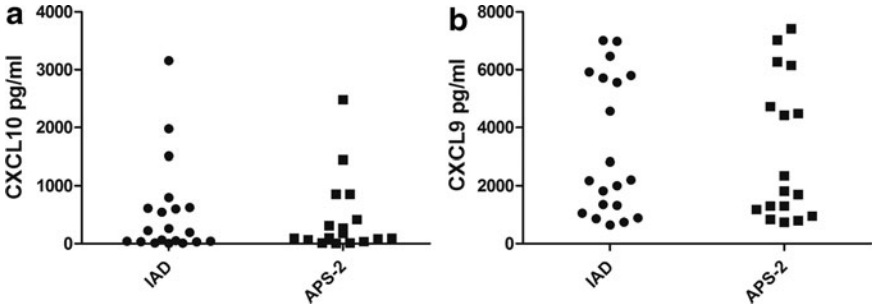

SUPPLEMENTARY FIG. S1. Variations in plasma and serum levels of CXCL10 **(a)** and CXCL9 **(b)** as seen in Fig. 1, stratified between patients with isolated Addison's disease (IAD) and patients with autoimmune polyendocrine syndrome type 2 (APS-2). Nonparametric Mann–Whitney *U*-test was used to test for statistical differences between the 2 groups, but none were found.
