# Supplementary material for: Peripheral Blood Cells from Patients with Autoimmune Addison's Disease Poorly Respond to Interferons In Vitro, Despite Elevated Serum Levels of Interferon-Inducible Chemokines
Source: J Interferon Cytokine Res. 2015 Oct 1;35(10):759–70. doi: 10.1089/jir.2014.0171 (PMC4589105; doi:10.1089/jir.2014.0171)
Supplement: Supplemental data [file Supp_Fig2.pdf]

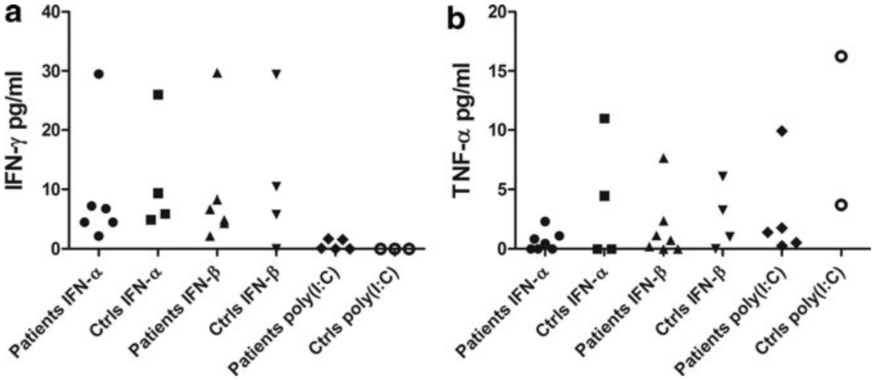

SUPPLEMENTARY FIG. S2. Production of IFN-γ **(a)** and TNF-α **(b)** in PBMC stimulated with type I IFNs and poly (I:C) in patients (IFN-γ: IFN-α, $n=6$; IFN-β, $n=6$; poly (I:C), $n=5$. TNF-α: IFN-α, $n=7$; IFN-β, $n=7$; poly (I:C), $n=5$) and controls (IFN-γ: IFN-α, $n=4$; IFN-β, $n=4$; poly (I:C), $n=3$. TNF-α: IFN-α, $n=4$; IFN-β, $n=4$; poly (I:C), $n=2$). Non-parametric Mann–Whitney *U*-test was used to test for statistical differences between patients and controls, but none were found. For poly (I:C) there were too few data points to do calculations. IFN, interferon; PBMC, peripheral blood mononuclear cell.
