# Supplementary material for: Peripheral Blood Cells from Patients with Autoimmune Addison's Disease Poorly Respond to Interferons In Vitro, Despite Elevated Serum Levels of Interferon-Inducible Chemokines
Source: J Interferon Cytokine Res. 2015 Oct 1;35(10):759–70. doi: 10.1089/jir.2014.0171 (PMC4589105; doi:10.1089/jir.2014.0171)
Supplement: Supplemental data [file Supp_Fig12.pdf]

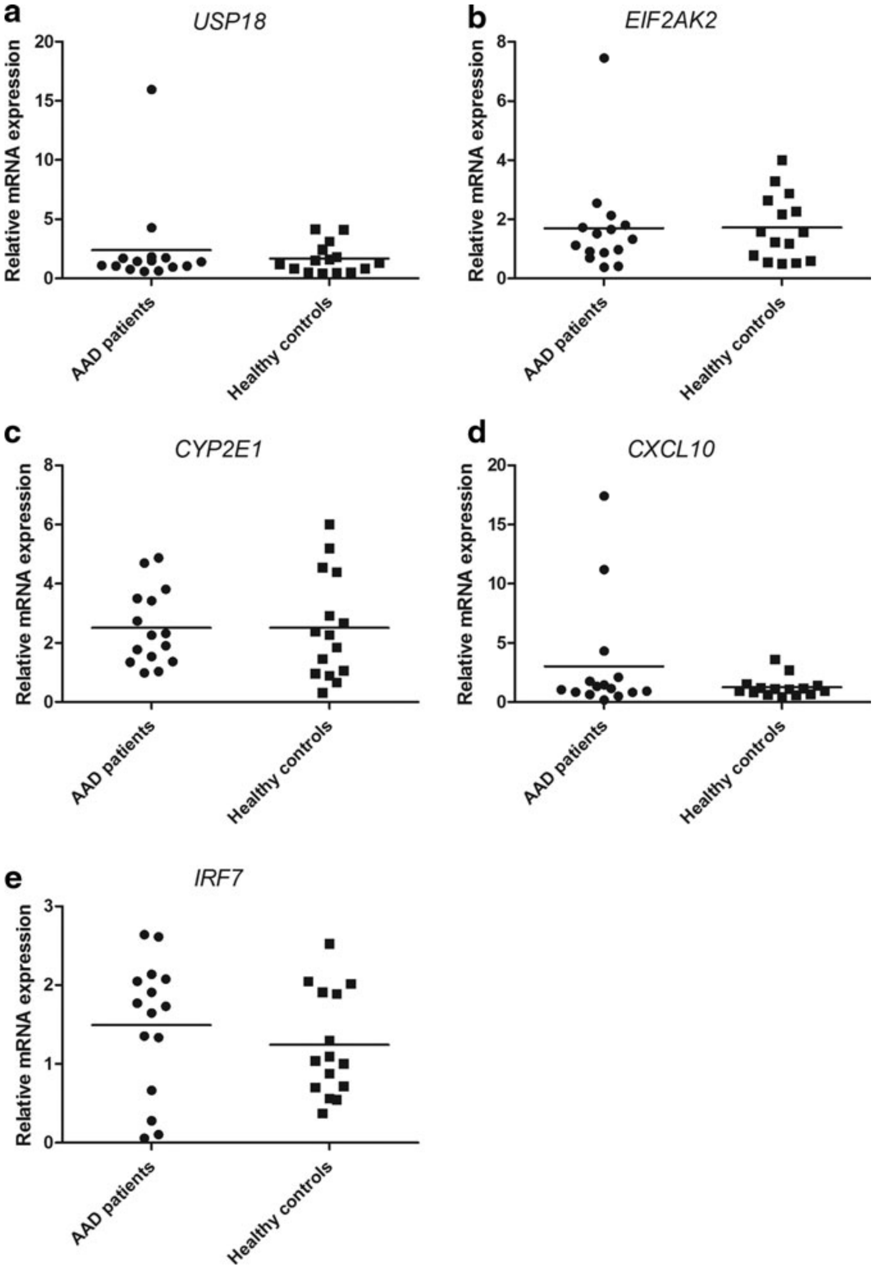

SUPPLEMENTARY FIG. S12. Real-time qPCR was used to determine the relative mRNA expression of 5 selected ISGs **(a–e)** in isolated CD4$^+$ T cells from AAD patients ($n = 15$) and healthy controls ($n = 15$). The results for each patient and control are displayed as means of triplicates and the bars display the mean for the whole group. Nonparametric Mann–Whitney *U*-test was used to test for statistical differences between patients and controls, but none were found. AAD, autoimmune Addison's disease; ISGs, interferon-stimulated genes; qPCR, quantitative real-time polymerase chain reaction.
