# Supplementary material for: Peripheral Blood Cells from Patients with Autoimmune Addison's Disease Poorly Respond to Interferons In Vitro, Despite Elevated Serum Levels of Interferon-Inducible Chemokines
Source: J Interferon Cytokine Res. 2015 Oct 1;35(10):759–70. doi: 10.1089/jir.2014.0171 (PMC4589105; doi:10.1089/jir.2014.0171)
Supplement: Supplemental data [file Supp_Fig14.pdf]

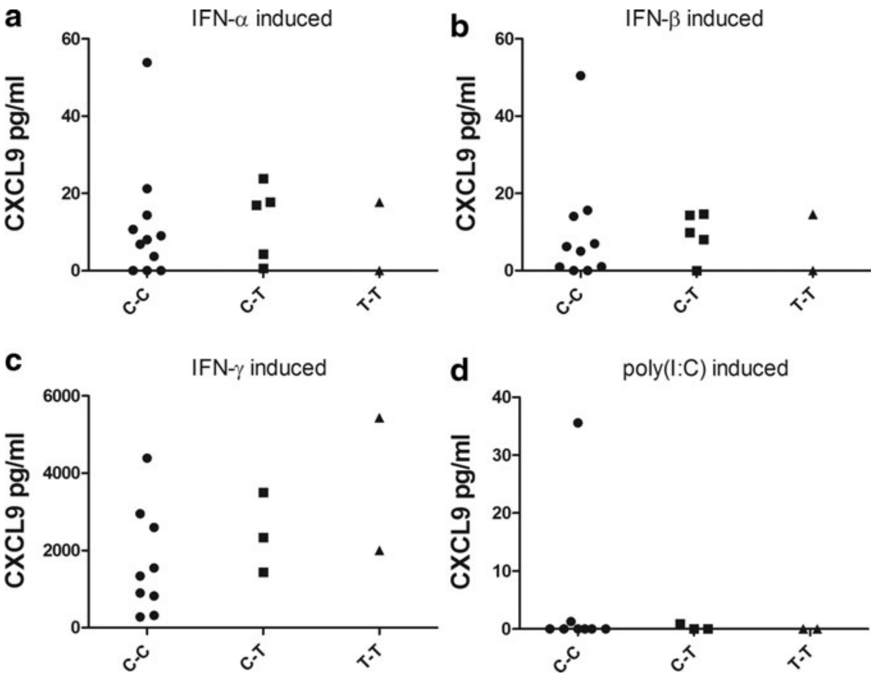

SUPPLEMENTARY FIG. S14. Variations in IFN- **(a–c)** and poly (I:C) **(d)**-induced CXCL9 production as seen in Figs. 2 and 4 stratified between patients with the 3 different PTPN22 1858 genotypes.
